# The Role of IRX Homeobox Genes in Hematopoietic Progenitors and Leukemia

**DOI:** 10.3390/genes14020297

**Published:** 2023-01-23

**Authors:** Stefan Nagel

**Affiliations:** Department of Human and Animal Cell Cultures, Leibniz-Institute DSMZ, 38124 Braunschweig, Germany; sna@dsmz.de; Tel.: +49-531-2616167

**Keywords:** B-ALL, hematopoiesis, homeodomain, leukemia, NKL, T-ALL

## Abstract

IRX genes are members of the TALE homeobox gene class and encode six related transcription factors (IRX1–IRX6) controlling development and cell differentiation of several tissues in humans. Classification of TALE homeobox gene expression patterns for the hematopoietic compartment, termed TALE-code, has revealed exclusive IRX1 activity in pro-B-cells and megakaryocyte erythroid progenitors (MEPs), highlighting its specific contribution to developmental processes at these early stages of hematopoietic lineage differentiation. Moreover, aberrant expression of IRX homeobox genes IRX1, IRX2, IRX3 and IRX5 has been detected in hematopoietic malignancies, including B-cell precursor acute lymphoblastic leukemia (BCP-ALL), T-cell ALL, and some subtypes of acute myeloid leukemia (AML). Expression analyses of patient samples and experimental studies using cell lines and mouse models have revealed oncogenic functions in cell differentiation arrest and upstream and downstream genes, thus, revealing normal and aberrant regulatory networks. These studies have shown how IRX genes play key roles in the development of both normal blood and immune cells, and hematopoietic malignancies. Understanding their biology serves to illuminate developmental gene regulation in the hematopoietic compartment, and may improve diagnostic classification of leukemias in the clinic and reveal new therapeutic targets and strategies.

## 1. Introduction

**Figure 1:**
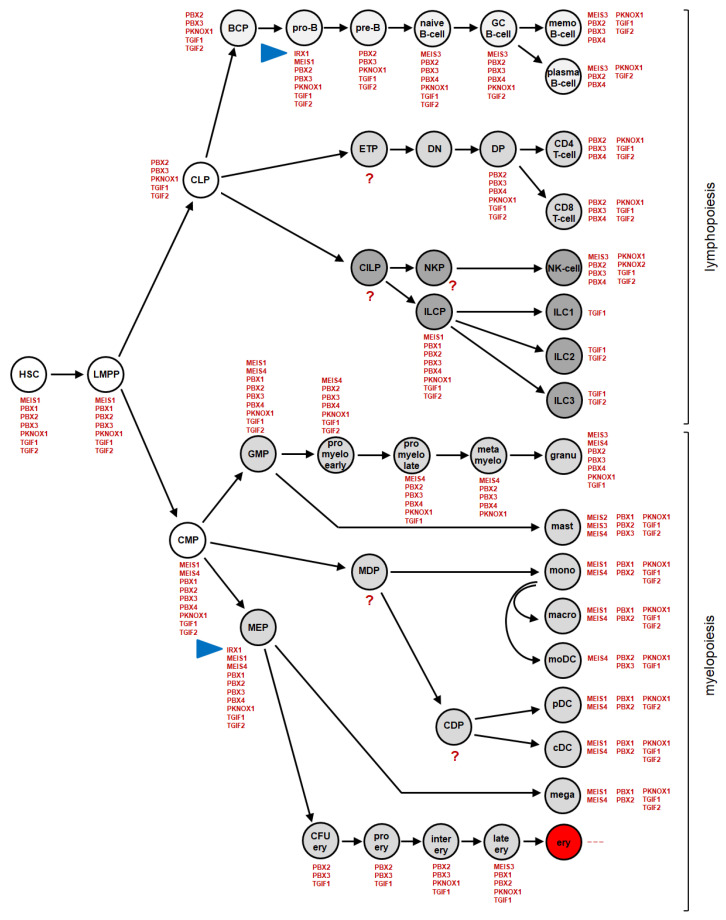
This diagram illustrates the expression patterns of TALE class homeobox genes in the course of hematopoiesis, termed TALE-code. Please note, IRX1 is exclusively expressed in pro-B-cells during lymphopoiesis and in megakaryocyte erythroid progenitors (MEPs) during myelopoiesis (blue arrowheads). Abbreviations: BCP: B-cell progenitor; cDC: conventional dendritic cell; CDP: common dendritic cell progenitor; CFU ery: colony forming unit for erythrocytes; CILP: common innate lymphoid progenitor; CLP: common lymphoid progenitor; CMP: common myeloid progenitor; DN: double negative thymocytes; DP: double positive thymocytes; ery: erythrocyte,; ETP: early T-cell progenitor; GC: germinal center; granu: granulocyte; HSC: hematopoietic stem cell; ILC: innate lymphoid cell; ILCP: innate lymphoid cell progenitor; inter ery: intermediate erythroid; late ery: late erythroid; LMPP: lymphoid myeloid primed progenitor; macro: macrophage; mast: mast cell; MDP: monocyte dendritic cell progenitor; mega: megakaryoycte; MEP: megakaryocyte erythroid progenitor; moDC: monocyte-derived dendritic cell; mono: monocyte; NK-cell: natural killer cell; NKP: natural killer progenitor; pDC: plasmacytoid dendritic cell; pro myelo: pro-myelocyte; meta myelo: meta-myelocyte.

Immune and blood cells belong to the hematopoietic system and originate from hematopoietic stem cells (HSC) located in the bone marrow. This complex and plastic system has been extensively characterized by cytological, genetic, immunological and molecular methods including novel single-cell sequencing techniques, revealing origin and parentage of progenitors and mature cells alike [1,2]. Common myeloid and common lymphoid progenitors (CMP and CLP) descend from HSCs and represent the founders of the myeloid and lymphoid lineages, respectively (Figure 1).

CLPs produce all types of lymphocytes, comprising B-cells, T-cells, natural killer (NK) cells and innate lymphoid cells (ILC). Early B-cell development takes place in the bone marrow, starting with the CLP-derived B-cell progenitor (BCP). BCPs differentiate into naïve B-cells via the pro-B- and pre-B-cell stages [3]. Naïve B-cells migrate from the bone marrow into lymphoid tissues including lymph nodes and spleen, to undergo differentiation via the stage of germinal center (GC) B-cells into memory B-cells and plasma cells. In contrast, CLP-derived early T-cell progenitors (ETP) migrate to the thymus to complete differentiation into CD4 and CD8 single-positive T-cells via the double negative (DN) and double positive (DP) stages [1]. 

CMPs produce all types of myeloid cells including the granulocyte macrophage progenitor (GMP)-derived types of granulocytes, namely, eosinophils, neutrophils and basophils. The megakaryocyte erythroid progenitor (MEP) produces megakaryocytes and erythrocytes via specific intermediates [4,5]. The myeloid cell panel additionally contains mast cells and monocytes, the latter able to differentiate into macrophages or monocyte-derived dendritic cells (moDC), while conventional and plasmacytoid DCs originate from a common progenitor. In contrast with developing lymphocytes, all types of myeloid cells mature in the bone marrow [1].

The main differentiation steps of hematopoiesis are controlled at the transcriptional level by diverse transcription factors (TFs) often described as master factors [6,7]. TAL1/SCL, LYL1 and TCF3/E2A are members of the basic helix–loop–helix protein family. While TAL1 and LYL1 regulate vital steps in early and late hematopoiesis, TCF3 is active in B- and T-cell differentiation [8]. During B-cell development, TCF3 activates expression of immunoglobulins in addition to the B-cell master factors EBF1 and PAX5 [9]. GATA factors are zinc-finger proteins that control hematopoietic stem cells (GATA2), erythropoiesis and megakaryopoiesis (GATA1), and NK- and T-cell development (GATA3) [10]. Additional master TFs controlling specific processes in hematopoietic differentiation are BCL11B (regulating development of T-cells), NFIL3 (NK-cells), ID2 (ILCs), and SPI1/PU.1 (myeloid cells) [11,12,13,14]. These developmental regulators belong to different protein families, demonstrating that various types of TFs perform transcriptional control of hematopoiesis.

Homeobox genes encode the second largest group of TFs in humans and regulate fundamental steps in development and differentiation, both in embryogenesis and in the adult [15,16]. Paralleling these physiological functions, deregulated homeobox genes underlie corresponding developmental disorders or contribute to carcinogenesis. Depending on the tumor type, homeobox genes may act as oncogenes or tumor suppressors [17,18]. These genes share the 180 bp long conserved homeobox that encodes the 60 amino acid residues containing homeodomain at the protein level. This domain forms three helices generating a specific 3D structure classified as helix–turn–helix, which performs specific interactions with DNA, chromatin and cooperating TFs [16]. Helix 3 fits into the major groove of the DNA and conducts sequence-specific interactions, while helices 1 and 2 stabilize the domain structure and, together with flanking amino acid residues, perform additional DNA contacts [19]. 

Systematic classification of all 235 human homeobox genes generates a panel of 11 classes and several subclasses [20]. One of the main classes, antennapedia (ANTP), contains the subclasses NKL and HOXL that include the clustered HOX genes. Other classes hitherto identified are CERS, CUT, HNF, LIM, POU, PRD, PROS, SINE, TALE, and ZF. TALE class homeobox genes comprise 20 members in humans and share a three amino acid loop extension between helix 1 and 2, abbreviated as TALE [20]. This ancient group of homeobox genes encodes TFs, which are able to cooperate with other TALE or particular HOX proteins to regulate target genes. The TALE class is divided into six groups, namely, IRX, MEIS, MKX, PBX, PKNOX and TGIF [21]. Here, we focus on IRX genes and their roles in normal and aberrant hematopoiesis. 

## 2. Homeobox Gene Codes

Homeobox genes share regulatory impacts on developmental processes. This statement is supported by the observation that identities of all 118 types of neurons in the nematode *Caenorhabditis elegans* are determined by specific homeobox genes, of which 102 are collectively present in the genome of this animal [22]. Furthermore, related homeobox genes are frequently involved in similar and evolutionarily conserved operations and functions. Thus, for example, HOX genes regulate the generation of anterior–posterior axes in embryos. These and similar observations were used to establish HOX-codes that represent signatures of all expressed HOX genes in the developing pharyngeal region, hindbrain or neural crest cells along this axis [23,24,25]. The DLX-code comprises DLX homeobox genes expressed in the pharyngeal region showing a dorso–ventral pattern, while specific PAX genes determine the identity of developing placodes [26,27]. Thus, homeobox gene codes consist of related members from a selected homeobox gene group that show a distinct expression pattern in a particular compartment of the body. We have applied this concept to the human hematopoietic system having focused on NKL subclass homeobox genes in addition to TALE class homeobox genes to generate the corresponding NKL- and TALE-codes [28,29,30,31,32]. In contrast with previously described homeobox gene codes, we have used these signatures to compare normal and malignant development. Thus, we evaluated particular leukemia/lymphoma entities according to their aberrant NKL and TALE homeobox gene activities [28,29,30,31,32]. Identified deregulated homeobox genes may be useful for classification and diagnostics of hematopoietic malignancies and even reveal novel therapeutic target genes implicated in altered networks. 

The actual TALE-code is depicted in Figure 1. It was established in two steps, by analyzing the respective myeloid and lymphoid lineages, the latter completed only recently [29,30,31]. According to these data, the human hematopoietic compartment expresses eleven TALE class homeobox genes, comprising IRX1, MEIS1, MEIS2, MEIS3, PBX1, PBX2, PBX3, PBX4, PKNOX1, TGIF1 and TGIF2. The numbers of expressed TALE homeobox genes in single entities range from three to nine. Furthermore, this code shows different types of gene expression patterns: TGIF genes are expressed in all but one entity; PBX1 expression is restricted to progenitor cells in the lymphoid lineage while also active in mature myeloid cell types; and IRX1 expression is restricted to pro-B-cells and MEPs. Thus, IRX1 is exclusively activated in two respective types of progenitors, belonging to the lymphoid and myeloid system. These observations indicate a role for IRX1 in normal hematopoiesis, while related IRX genes may have an impact on hematopoietic malignancies following deregulation. 

## 3. IRX1 Expression and Regulation in Hematopoiesis

### 3.1. IRX1 in Lymphopoiesis

IRX1 is a founding member of the six IRX genes reported in vertebrates [33,34,35]. Their names derive from the Iroquois homeobox gene complex (Iro-C), identified in *Drosophila*. Iro-C consists of two TALE homeobox genes, namely, Araucan and Caupolican [36]. Fruit flies carrying a mutant Iro-C locus have lost lateral bristles of their notum, recalling the hairstyle ascribed to the Iroquois tribe [37]. In the human genome, IRX1 is arranged in a cluster together with IRX2 and IRX4 at chromosomal position 5p15 while IRX3, IRX5 and IRX6 are clustered at 16q12. 

**Figure 2:**
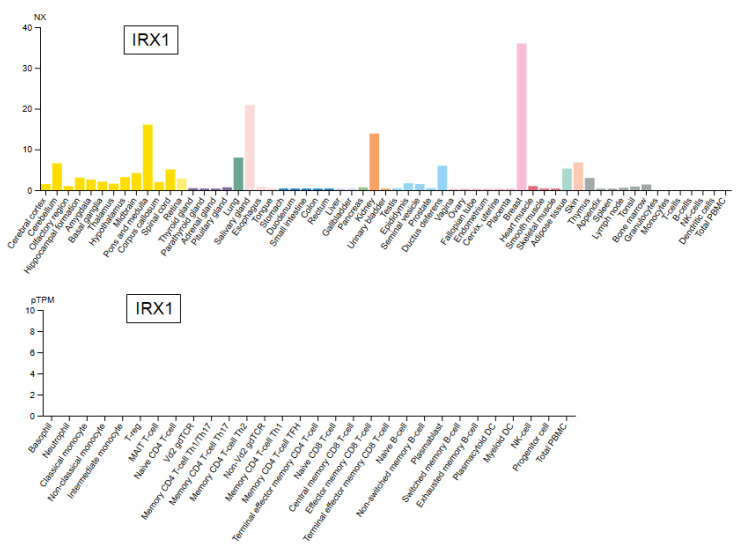
RNA-seq based expression data for IRX1 derived from the Human Protein Atlas. Elevated IRX1 levels are detectable in the brain, lung, salivary gland, kidney and breast (**above**) while selected immune and blood cells do not express IRX1 (**below**).

IRX genes encode TFs which differ in their function due to sequence disparities and tissue-specific activities. According to RNA-seq data from the Human Protein Atlas [38], IRX1 is expressed in several tissues, including brain, lung, salivary gland, kidney and breast, but remain silent in mature blood and immune cells (Figure 2). Functionally, IRX1 is implicated in kidney development and in neuronal differentiation, corresponding to its expression profile [39,40]. Thus, IRX1 encodes a TALE class homeodomain TF, regulating tissue-specific developmental processes. 

**Figure 3:**
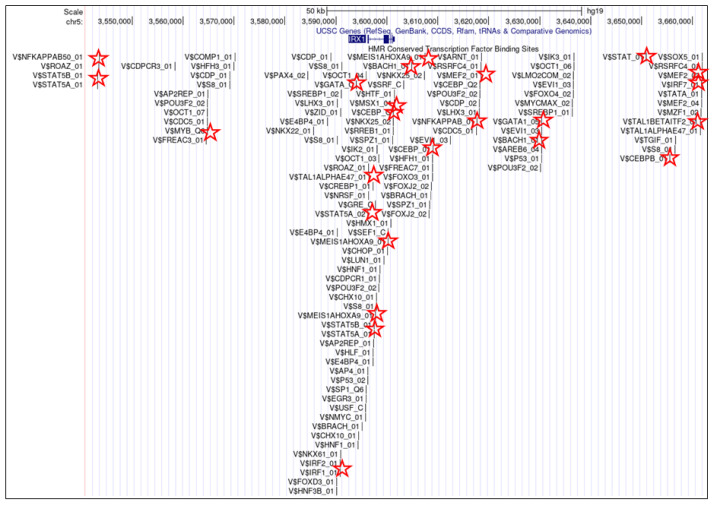
This diagram was obtained from the UCSC genome browser and shows just 100 kb of the about 2 Mb long regulatory region of IRX1 (coding part in blue). It contains several potential binding sites for TFs, including hematopoietic TFs such as BACH, CEBP, GATA, IRF, MEF2, MEIS-HOXA, MSX1, MYB, NFkB, STAT, STAT5, TAL1 and TCF3/E47 (indicated by red asterisks). All these factors represent potential regulators, while GATA1, GATA2 and TCF3 are confirmed activators of IRX1 transcription.

The establishment of the lymphoid TALE-code revealed expression of IRX1 exclusively in pro-B-cells [31]. Thus, during B-cell differentiation, IRX1 is activated in this progenitor to perform specific developmental operations. The extensive genomic IRX1 regulatory region covers about 2 Mb, indicating the need for detailed control of this gene, typical for developmental master regulators. Inspection of the IRX1 gene locus using data from the UCSC genome browser revealed numerous potential TF binding sites, including several for hematopoietic TFs, such as GATA and TCF3 (Figure 3). Accordingly, transcriptional analysis of IRX1 performing siRNA-mediated knockdown in human leukemic B-cell lines demonstrated that TCF3 is an activating factor [31]. TCF3 encodes two alternatively spliced TFs of the basic helix–loop–helix family, called E12 and E47, both master factors in lymphopoiesis and B-cell development [8,9]. Additional knockdown experiments showed that IRX1, in turn, drives expression of TCF3, indicating that IRX1 and TCF3 are mutual activators [31]. This interpretation is supported by elevated expression levels of both genes in pro-B-cells [31]. Taken together, IRX1 forms part of a physiological gene regulatory network driving B-cell differentiation. 

In addition, IRX1 is physiologically expressed in several non-hematopoietic tissues such as lung (Figure 2). However, in lung adenocarcinoma, IRX1 expression is suppressed by DNA hypermethylation while hypomethylation mediates its aberrant activation in osteosarcoma [41,42]. These data show that DNA-methylation represents an important mechanism of IRX1 regulation which may also play a role in the hematopoietic compartment. 

### 3.2. IRX1 in Myelopoiesis

**Figure 4:**
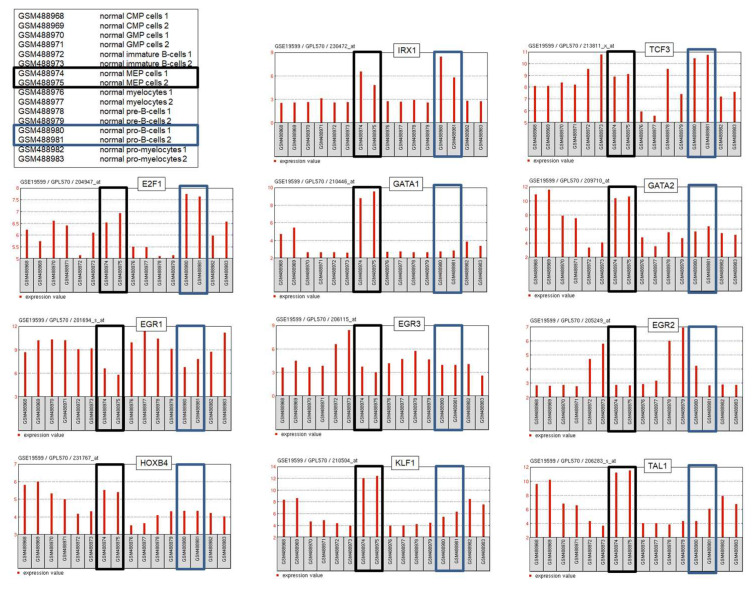
Expression data obtained from GEO (dataset GSE19599) for IRX1, E2F1, TCF3, GATA1, GATA2, EGR1, EGR2, EGR3 and HOXB4 from hematopoietic progenitor cells including pro-B-cells (boxed in blue) and MEPs (boxed in black). Elevated or reduced expression levels in pro-B-cells and/or MEPs support regulatory interactions of these genes/factors.

In addition to lymphoid pro-B-cells, IRX1 shows activity in the myeloid MEPs [30]. GATA factors are closely related TFs containing two Zn-finger DNA binding domains which recognize cognate DNA-sites [43]. Three of these, GATA1, GATA2, and GATA3, represent fundamental hematopoietic TFs. GATA1 and GATA2 are basic regulators of myelopoiesis; GATA2 plays important roles in stem and progenitor cells and is substituted by GATA1 in subsequent differentiation steps [10,43]. MEPs (but not pro-B-cells) express elevated levels of both GATA1 and GATA2 (Figure 4), marking transmission from progenitor to more differentiated cell types. Knockdown experiments in myeloid cell lines demonstrated that both GATA1 and GATA2 mediate transcriptional activation of IRX1 [30], consistent with the presence of potential GATA binding sites at the IRX1 locus (Figure 3). Thus, activation of IRX1 transcription in MEPs is probably performed by the master factors GATA1 and GATA2. Additional studies using myeloid and non-hematopoietic cell lines revealed that IRX1, in turn, regulates expression of KLF1, TAL1, EGR1/2/3 and HOXB4 [30,44]. Thus, physiological IRX1 is probably embedded in a network consisting of myeloid master TFs that show specific activities in MEPs (Figure 4). 

Taken together, IRX1 is physiologically expressed in specific hematopoietic progenitors, namely, pro-B-cells and MEPs. Co-expression data from normal progenitor cells and experimental results from cell lines indicates that IRX1 forms an integral part of lymphoid and myeloid gene regulatory networks. To date, the lymphoid network consists of IRX1 and TCF3 while the myeloid network contains IRX1, EGR1/2/3, GATA1/2, HOXB4, KLF1 and TAL1. 

## 4. Aberrant IRX Gene Activities in Hematopoietic Malignancies

### 4.1. Deregulated IRX Genes in Acute Lymphoid Leukemia

According to the lymphoid TALE-code, IRX1 is exclusively expressed in pro-B-cells [31]. B-cell precursor acute lymphoblastic leukemia (BCP-ALL) originates from aberrant early B-cell progenitors including pro-B-cells and shows differentiation arrest at these stages. This type of leukemia is predominant in children and is classified according to the presence of specific fusion genes [45,46,47,48,49]. Evaluation of public BCP-ALL data for deregulated IRX genes reveals aberrant expression of IRX1, IRX2 and IRX3 in subsets of patients [31]. Moreover, these genes show a specific expression pattern according to karyotype markers. IRX2 expression correlates with the presence of TCF3-fusions and IRX3 with ETV6-fusions [31,50]. In contrast, IRX1 shows no specific correlation, indicating residual activity according to the corresponding early stage of B-cell development [31]. Furthermore, aberrant activation of IRX3 has also been reported in 48% of T-cell ALL patients, correlating with HOXA gene activities [50].

**Figure 5:**
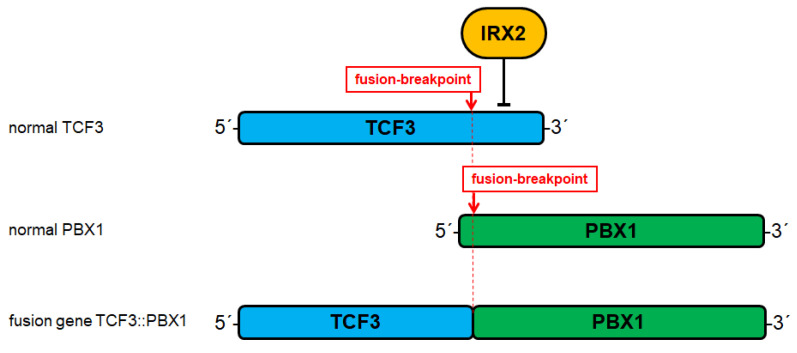
Deregulation of TCF3 in BCP-ALL. TCF3 is frequently mutated by chromosomal translocation resulting in gene fusions with particular partners including PBX1. The fusion breakpoints and the generated TCF3::PBX1 fusion gene are indicated. IRX2 binds at intron 18 of TCF3 and suppresses its transcription. The genomic fusion of TCF3 and PBX1 deletes this inhibitory IRX2 binding site.

BCP-ALL cell lines aberrantly expressing IRX genes serve as suitable models to analyze their regulatory networks. Expression data for E2F1 from normal pro-B-cells and malignant BCP-ALL cell lines demonstrate elevated transcript levels (Figure 4), indicating that E2F1 may represent a physiological regulator of IRX1 in these progenitors [31]. However, knockdown experiments performed in BCP-ALL cell lines together with ChIP-seq data from the ENCODE database (dataset GSM935477) show that E2F1 activates IRX2 but not IRX1 via direct interaction at the IRX2 locus [31]. BCP-ALL cell line 697 expresses IRX2 and carries fusion gene TCF3::PBX1. Experiments using this cell line model have revealed that IRX2 inhibits wild type TCF3 but not TCF3::PBX1 [31]. An IRX2 binding site identified at intron 18 is deleted in the fusion gene (Figure 5). Thus, IRX2 is unable to bind and inhibit TCF3::PBX1. TCF3 encodes an important factor for B-cell development that is disturbed by gene fusions, indicating that it is a tumor suppressor [8,31]. Downregulation of TCF3 but not of oncogenic TCF3::PBX1 represents an additional mechanism of deregulated B-cell development via TCF3 perturbation.

**Figure 6:**
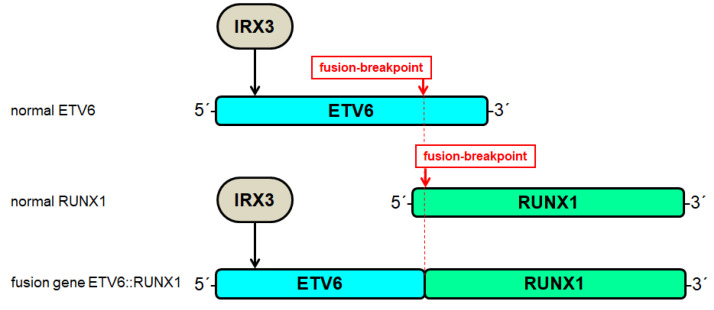
Deregulation of ETV6 in BCP-ALL. ETV6 is frequently mutated by chromosomal translocation resulting in gene fusions with particular partners including RUNX1. The fusion breakpoints are indicated. IRX3 binds at intron 1 of ETV6 and activates its transcription. The genomic fusion of ETV6 and RUNX1 maintains this activating IRX3 binding site.

BCP-ALL cell line REH expresses IRX3 and carries fusion gene ETV6::RUNX1. Paralleling the situation in 697 cells, IRX3 binds ETV6 at intron 1. However, IRX3 activates expression of ETV6::RUNX1 because the fusion event maintains this binding site (Figure 6). Thus, IRX3 performs activation of both, wild type ETV6 and fusion gene ETV6::RUNX1 [31]. However, ETV6 encodes a developmental B-cell factor that also operates as a tumor suppressor [51]. Therefore, deletion of ETV6 in IRX3-positive cells possibly serves to escape transcriptional activation by this homeodomain factor [31]. Sequencing data from T-ALL cell lines show aberrant expression exclusively of IRX3 while the remaining IRX genes are silent, indicating functional differences between IRX oncogenes in B-cell and T-cell ALL [31]. In T-ALL, oncogenic TAL1 interacts and cooperates with TCF3 to deregulate target genes [52,53]. Therefore, IRX2-mediated repression of TCF3 may have an adverse effect in TAL1-positive T-ALL. 

Taken together, BCP-ALL and T-ALL patients and cell lines aberrantly express IRX homeobox genes IRX1, IRX2 and IRX3. IRX1 expression in BCP-ALL may reflect its normal activity in pro-B cells in which this type of leukemia arises. The occurrence of IRX2 and IRX3 correlates with particular fusion genes in BCP-ALL and supports their oncogenic activity. IRX3 may represent the only deregulated IRX gene in T-ALL, cooperating with HOXA genes. IRX genes, thus, comprise novel oncogenes and may serve as diagnostic markers in ALL.

### 4.2. Deregulated IRX Genes in Acute Myeloid Leukemia

The establishment of the myeloid TALE-code revealed expression of IRX1 exclusive to MEPs [30]. Accordingly, screening for aberrant IRX gene activities in acute myeloid leukemia (AML) revealed overexpression of IRX1, IRX3 and IRX5 in subsets of patients, identifying these genes as oncogenes for this type of malignancy [30]. Aberrant IRX gene expression in AML has been identified by other approaches as well. HOXA genes are activated by KMT2A-fusion proteins and act as oncogenes in AML. However, the observation of reduced HOXA gene expression levels in patients containing fusion protein KMT2A::AFF1 led to the identification of IRX1, which inhibits KMT2A::AFF1 activity by direct interaction [44]. Of note, this differential expression pattern of HOXA and IRX genes has been reported in ALL as well [54,55]. In contrast, elevated expression of IRX3 correlated with oncogene FOXC1 in HOXA-positive AML analyzed in detail in an additional study by the same investigators [56]. Hence, functional analysis of IRX3 in AML revealed inhibition of myelomonocytic differentiation [50]. Thus, aberrantly expressed IRX factors in myeloid progenitors are involved in developmental arrest and correlate with HOXA gene activity.

**Figure 7:**
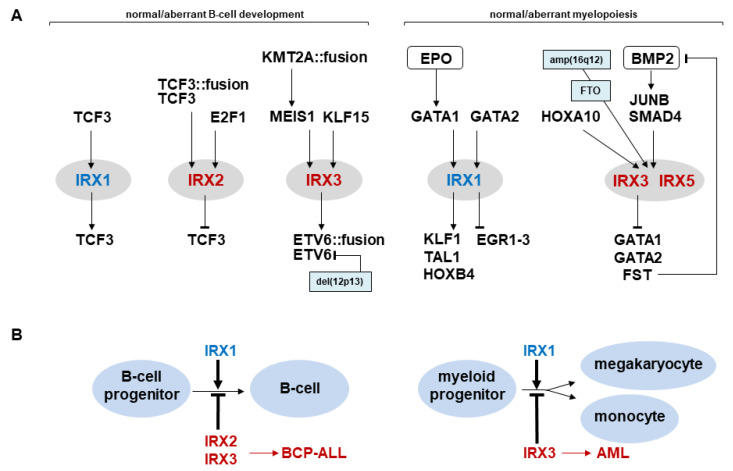
IRX genes in normal and aberrant hematopoiesis. (**A**) Upstream regulators and downstream target genes of IRX factors in B-cell development (left) and myelopoiesis (right). (**B**) IRX1 drives normal differentiation of B-cells (left) and myeloid cells (right) while aberrantly expressed IRX1, IRX2 and IRX3 deregulate cell differentiation mediating developmental arrest and leukemogenesis.

Identified AML cell lines expressing IRX genes serve as models to analyze their regulatory networks. The data show that aberrantly expressed IRX3 is activated by HOXA10 and BMP2-signaling via JUNB and SMAD4 [30]. In megakaryoblastic AML cell line MEGAL, neighboring IRX3 and IRX5 loci are focally amplified and overexpressed together with FTO [30]. FTO contains an enhancer for neighboring IRX genes and plays a role in obesity and cancer, including AML [57,58,59,60]. Thus, genomic aberrations of these loci may underlie IRX activation in subsets of this malignancy. In contrast, IRX3 itself operates as an inhibitor, suppressing transcription of GATA1, GATA2 and FST [30]. FST is an inhibitor of BMP2—its repression, thus, boosts activating BMP2-signaling, thereby generating a feedback loop (Figure 7). Recently, BMP2 has been shown to play an abnormal role in self-renewal of MEPs coinciding with its function in oncogenic IRX3 activation [61]. Downregulation of GATA1/2 may contribute to the reported differentiation arrest, since GATA factors perform major developmental operations in myelopoiesis [10,43].

The OMIM database (www.omim.org, accessed on 11 January 2023) provides disease related information for all six IRX genes, namely IRX1: OMIM 606197, IRX2: OMIM 606198, IRX3: OMIM 612985, IRX4: OMIM 606199, IRX5: OMIM 606195, and IRX6: OMIM 606196. Knockout mice models were listed in the MGI database (www.informatics.jax.org, accessed on 11 January 2023) for IRX1, IRX3, IRX5 and IRX6. However, these resources did not refer to hematopoietic/immunological diseases or functions for any IRX gene to date.

Taken together, both AML patients and cell lines which aberrantly express IRX homeobox genes IRX1, IRX3 and IRX5 have been reported, reflecting their contribution to regulatory networks controlling myelopoiesis and driving differentiation arrest (Figure 7). The study of these genes may be employed to support clinical diagnostics for AML and, together with identification of further target genes, may reveal novel therapeutic strategies in the future.

## 5. Conclusions

IRX genes are closely related members of the TALE homeobox gene class which differ in their sequence and tissue-specific activity. IRX1 is physiologically expressed in pro-B-cells and MEPs and part of corresponding regulatory networks. Aberrant activities of IRX genes contribute to the development of hematopoietic malignancies including BCP-ALL, T-ALL and AML via deregulation of developmental processes (Figure 7). Aberrantly expressed IRX genes may serve as additional diagnostic markers in the clinic and characterization of their target genes may assist the development of novel targeted therapies.

## Data Availability

No new data were created or analyzed in this study. Data sharing is not applicable to this article.
